# Accuracy of a point-of-care CoaguChek test versus standard laboratory coagulation monitoring in cardiac surgery involving cardiopulmonary bypass: randomized clinical trial

**DOI:** 10.1016/j.bjane.2025.844714

**Published:** 2025-11-22

**Authors:** Fabrício Tavares Mendonça, Sérgio Honorato de Matos, Larissa Goveia Moreira, Igor Louza Pereira, Lorenzo Leite Dino, Matheus Beserra Braga, Gustavo Henrique dos Santos Dias

**Affiliations:** Hospital de Base do Distrito Federal, Departamento de Anestesiologia, Brasília, DF, Brasil

**Keywords:** Blood coagulation tests, Cardiopulmonary bypass, Diagnostic, point of care, International normalized ratio

## Abstract

**Background:**

Intraoperative coagulopathies are common in complex surgeries, and timely coagulation monitoring is crucial. We assessed the accuracy of the point-of-care CoaguChek XS test against standard laboratory measurements in patients undergoing cardiac surgery with Cardiopulmonary Bypass (CPB).

**Methods:**

We conducted a single-center, diagnostic accuracy study to assess the coagulation profile of 50 participants before and after CPB. The index test was the CoaguChek XS device and the reference test was the standard laboratory assay. The primary outcome was the accuracy of the CoaguChek device in measuring the International Normalized Ratio (INR). We pre-specified a tolerance range of ± 0.5 INR units. Secondary outcomes included accuracy in measuring prothrombin time and prothrombin activity.

**Results:**

We included 50 patients undergoing cardiac surgery with CPB between October 2023 and January 2024. The mean (standard deviation) age was 59.2 (12.3) years, and 32 participants (64%) were male. For INR values, Lin’s coefficient was 0.72 (95% CI: 0.60‒0.82) pre-CPB and 0.66 (95% CI: 0.50‒0.77) post-CPB, both indicating good agreement. In the pre-CPB period, on average, the index test readings exceeded reference readings by 0.045 INR units (95% CI: 0.030‒0.059, p < 0.001), while in post-CPB period, index readings were, on average, 0.064 INR units lower (95% CI: -0.09 to -0.04, p < 0.001). Although statistically significant differences were observed, they fell within predefined tolerance range and were considered clinically irrelevant. Analyses of secondary outcomes were consistent with the primary outcome findings.

**Conclusion:**

In patients undergoing cardiac surgery with cardiopulmonary bypass, CoaguChek XS provided results comparable to standard laboratory coagulation monitoring, both pre-and post-cardiopulmonary bypass.

**Research Board Approval:**

Plataforma Brasil CAAE 70266023.8.0000.5553.

**Clinical Trials Registration:**

(https://clinicaltrials.gov/study/NCT06037720) NCT06037720, 14/09/2023.

## Introduction

Effective monitoring of blood coagulation is essential in perioperative care, particularly during surgeries with a high risk of bleeding. While intraoperative fluid replacement is often necessary, it can dilute coagulation factors, potentially worsening bleeding and increasing the need for transfusions. This dynamic highlights the need for improved, rapid coagulation assessment methods.^1^ The Prothrombin Time (PT) test is used to diagnose and manage bleeding, and it can be performed either through standard laboratory tests or point-of-care devices. PT assesses the integrity of the extrinsic and common coagulation pathways. The International Normalized Ratio (INR), calculated from the PT, standardizes results across laboratories, regardless of reagents used.^2^

Point-of-care coagulation tests have been recommended in the perioperative setting.^3^ However, in Brazil, standard laboratory assays remain more commonly used due to their wider availability and lower cost, despite their longer turnaround times.^4^ Regarding the devices licensed in Brazil, CoaguChek devices have been recommended for the follow-up of patients after cardiac surgery, due to their practicality.^5^^,^^6^ Nevertheless, the accuracy of Coaguchek compared with standard laboratory methods for PT and INR measurement in cardiac surgery, especially in patients undergoing cardiopulmonary bypass, has not been well established.^7^^,^^8^

In this study, our general objective was to evaluate the accuracy and agreement between the CoaguChek XS device and traditional laboratory methods. Our specific objectives were to assess the performance of the point-of-care test in measuring INR, prothrombin time, and prothrombin activity in Brazilian patients undergoing elective cardiac surgery with cardiopulmonary bypass.

## Methods

### Study design

This was a prospectively planned, single-center, cross-sectional, diagnostic accuracy study conducted between October 01, 2023, and January 10, 2024. The study was conducted at the Hospital de Base do Distrito Federal, Brasília, Brazil, a tertiary, public reference center for cardiac surgeries. All methods were pre-specified (ClinicalTrials.gov registration: NCT06037720). The study was approved by the local Research Ethics Committee (Research Ethics Committee of the Strategic Health Management Institute of the Federal District) on August 10, 2023, under approval number 6.232.031, CAAE 70266023.8.0000.5553. All participants provided written informed consent. We adhered to the STARD guidelines for diagnostic accuracy studies to report our results.^9^

### Participants

We included consecutive participants of both sexes aged 18 years or older, undergoing elective cardiac surgery (coronary artery bypass grafting, valve replacement, and aortic surgery) with cardiopulmonary bypass. To be included, participants had to have an American Society of Anesthesiologists (ASA) physical status classification of I to III and baseline hemoglobin levels greater than 10 g.dL^-1^. Patients with liver or hematological diseases, conditions affecting coagulation (hemophilia, factor VII deficiency and others), or those participating in other studies were excluded.

### Test methods

#### Index test

The index test was the portable CoaguChek XS device (Roche, Switzerland), which uses an International Sensitivity Index (ISI) value close to 1.0 and measures the international normalized ratio within an analytical range of 0.8 to 8.0. A single device was used throughout the study, and it was pre-calibrated by the manufacturer; no additional calibration was required. All measurements were performed according to the manufacturer's instructions by trained investigators. The device's readings were obtained without knowledge of the reference test results. All measurements were successfully completed, and no invalid or indeterminate results occurred.

#### Reference test

The reference test was a standard laboratory-based assay conducted by a central hospital laboratory, using the same sample as the index test. All measurements were performed at the same facility, which is accredited by the Brazilian Society of Clinical Pathology/Laboratory Medicine (SBPC/ML) and adheres to relevant local standards and guidelines. Laboratory personnel were blinded to clinical information and index test results. According to the laboratory´s internal quality protocol, samples were processed immediately after collection, and the interval between blood collection and the reference assay did not exceed 30 minutes. the collection tubes were specifically designed to maintain sample integrity for coagulation testing during this period. All measurements were successfully completed, and no invalid or indeterminate results occurred.

### Procedures

After the induction of anesthesia (Web-Appendix 1), trained phlebotomists drew venous blood samples (03 mL) using standard techniques into 0.5 mL (3.2%) sodium citrate tubes. The samples were used to measure Activated Clotting Time (ACT) and blood gases. From the same sample, Prothrombin Time (PT) and International Normalized Ratio (INR) were assessed using both the index and reference tests. All tests were repeated 10 minutes after protamine administration and after the completion of the cardiopulmonary bypass. Blinding to the standard laboratory assay results was ensured by performing the index test immediately after blood collection, and before the reference test results were available.

### Outcomes

Our primary outcome was the accuracy of the index test in measuring the International Normalized Ratio (INR) compared with the standard laboratory method. We pre-specified a tolerance range of ± 0.5 INR units. This cutoff has been used in prior validation studies of point-of-care INR devices and aligns with recommendations that differences ≤ 0.5 INR units do not typically alter clinical management.^10^ Secondary outcomes included the agreement between index and reference measurements of Prothrombin Time (PT, in seconds) and prothrombin activity (PA, in %). Agreement between the index and reference tests was assessed both pre- and post-operatively to capture performance under distinct coagulation states. These time points reflect baseline and altered hemostasis conditions, ensuring a comprehensive evaluation of test accuracy. Central laboratory outcome measurements were blinded to participant status, study objectives, and prior point-of-care results.

### Sample size

The sample size was determined based on the primary outcome during the post-cardiopulmonary bypass period. This decision was informed by pilot data from 26 patients at the same center, which showed a larger standard deviation for the difference between the index and standard tests in the post-cardiopulmonary bypass period compared to the pre-cardiopulmonary bypass period. By focusing on the period with the greatest variation, we ensured that the final sample size was adequately powered in both periods. Agreement between the index and reference tests was assessed both pre- and post-operatively to capture performance under distinct coagulation states. These time points reflect baseline and altered hemostasis conditions, ensuring a comprehensive evaluation of test accuracy. For the calculations, we used the Bland-Altman limits of agreement, following the method developed by Lu et al. (2016). This test is a statistical method used to simultaneously assess the agreement between two measurement methods by evaluating both the mean and variance differences. Based on previous studies and our pilot data, we assumed a mean difference between the index test and the laboratory assay of -0.126 units, with a standard deviation of 0.132. Additionally, based on clinical criteria, we considered that an absolute difference greater than 0.5 unit would be clinically relevant. In other words, the maximum allowable difference (Δ) that would not affect clinical management was ± 0.5 units (i.e., differences within the range of -0.5 to 0.5 were considered clinically irrelevant). Assuming a 5% significance level (α) and 95% Confidence Intervals around the Bland-Altman limits of agreement, 43 participants were required to ensure 90% statistical power to demonstrate agreement between the two tests. To account for a potential 15% dropout or missing data rate, we increased the final sample size to 50 patients. This adjustment was made a priori, before data collection began, to preserve statistical power in case of incomplete observations. No interim or adaptive analyses were performed, and all data were analyzed after study completion.

### Statistical analysis

We conducted all statistical analyses following the Guidelines for Reporting Reliability and Agreement Studies and the recommendations by Gerke (2020).^11^^,^^12^ We presented continuous variables with approximately normal distributions as mean (Standard Deviation, SD), and those with a skewed distribution as median (Interquartile Range, IQR). Categorical variables were expressed as numbers (percentages).

The index and the reference tests were compared graphically using Bland-Altman plots. We assessed the normality of the differences between tests through graphical inspection. We used Lin's concordance correlation coefficient to evaluate the statistical agreement between the tests. This coefficient ranges from -1 to +1, with higher absolute values indicating stronger agreement. For Lin's coefficient (absolute values), we adopted the following pre-specified criteria:^13^ ≤ 0.4 indicates poor agreement, 0.41–0.60 moderate agreement, 0.61–0.80 good agreement, and > 0.81 excellent agreement. The cut-offs recommended by McBride were deemed less applicable to the clinical context of the present study.^14^ To assess whether the range of measurements was sufficiently broad, we applied the Preiss-Fisher procedure with 10,000 random resamplings.

The Bradley-Blackwood test was used to evaluate the global hypothesis of differences in means and/or variances between the two tests. When the Bradley-Blackwood test was statistically significant, we used Student's paired *t*-test to determine if the index and reference tests differed in their mean measurements. Subsequently, the Pitman-Morgan test was applied to assess whether the index and reference tests differed in their variability. All analyses were conducted using Stata (version 18, StataCorp, College Station, USA); p-values < 0.05 (two-tailed) were considered statistically significant.

## Results

**Figure 1:**
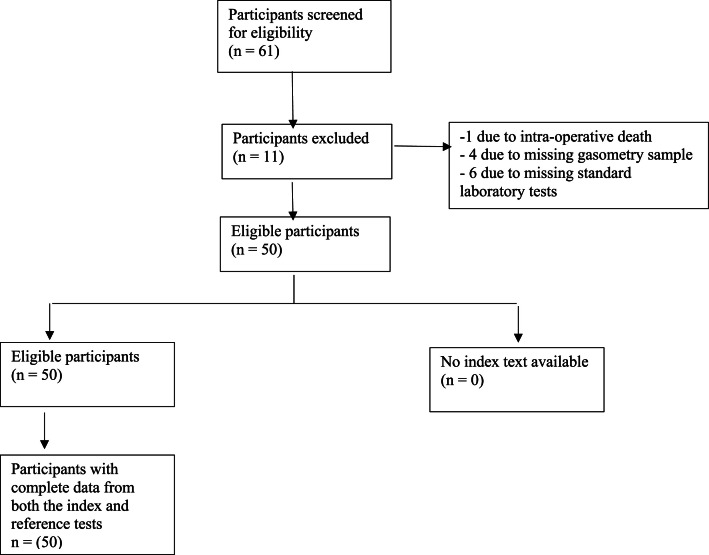
Flowchart summarizing the study selection process.

Between October 01, 2023, and January 10, 2024, we assessed 61 consecutive patients undergoing cardiac surgery with cardiopulmonary bypass, of whom 50 were included in the final analysis. All measurements yielded valid results for both the index and reference tests, with no indeterminate or invalid readings observed. Figure 1 shows details of the study selection process. Table 1 summarizes the main clinical and demographic characteristics of the 50 included participants. The mean age (SD) was 59.2 (12.3) years, and 32 participants (64%) were male. The most common diagnosis was angina, affecting 18 participants (36%).

**Table 1:** Baseline characteristics of participants, and biochemical and hematological parameters during the pre- and post-cardiopulmonary bypass periods (n = 50).

Variable	Estimate
Age, mean (SD)	59.2 (12.3)
Female sex, n (%)	32 (64)
Body mass index (kg.m^-2^), mean (SD)	26.3 (4.5)
Arterial hypertension, n (%)^a^	38 (81%)
Ejection fraction (%), mean (SD)^b^	56.3 (14.5)
Initial body temperature (°C), mean (SD)¶	
Hypothermia, n (%)	
Mild	42 (84%)
Moderate	8 (16%)
Clamp time (minutes), median (IQR)	92 (72 to 120)
Cardiopulmonary bypass time (minutes), median (IQR)	108.5 (88 to 148)
Pre-cardiopulmonary bypass period	
pH, mean (SD)	7.36 (0.05)
Bicarbonate, median (IQR)	23.4 (22.2 to 25.7)
Base excess, median (IQR)^a^	-1.7 (-2.5 to -0.3)
Hemoglobin, median (IQR)	11 (9.9 to 12.4)
Hematocrit, median (IQR)	34.9 (32 to 39.5)
Glucose, median (IQR)	111.5 (96.9 to 139)
Lactate, median (IQR)	1.39 (1.09 to 1.9)
Post-cardiopulmonary bypass period	
pH, mean (SD)	7.32 (0.05)
Bicarbonate, median (IQR)	21.9 (20.4 to 23.8)
Base excess, median (IQR)^a^	-3.85 (-5.3 to -2.2)
Hemoglobin, median (IQR)	9.3 (8.9 to 10.1)
Hematocrit, median (IQR)	28.8 (27.2 to 31.3)
Glucose, median (IQR)	142 (128 to 62)
Lactate, median (IQR)	3.48 (2.29 to 4.35)

IQR, Interquartile Range; SD, Standard Deviation.

a Based on 47 participants (3 with missing data).

b Based on 49 participants (1 with missing data). At the onset of the cardiopulmonary bypass period.

All graphical assessments revealed no major concerns regarding the normality of the differences between the index and reference tests. The Preiss-Fisher procedure confirmed that the measurement range was sufficiently wide for all outcomes (Web-Appendix 2).

### Agreement between the index and reference test – Pre-cardiopulmonary bypass

**Figure 2:**
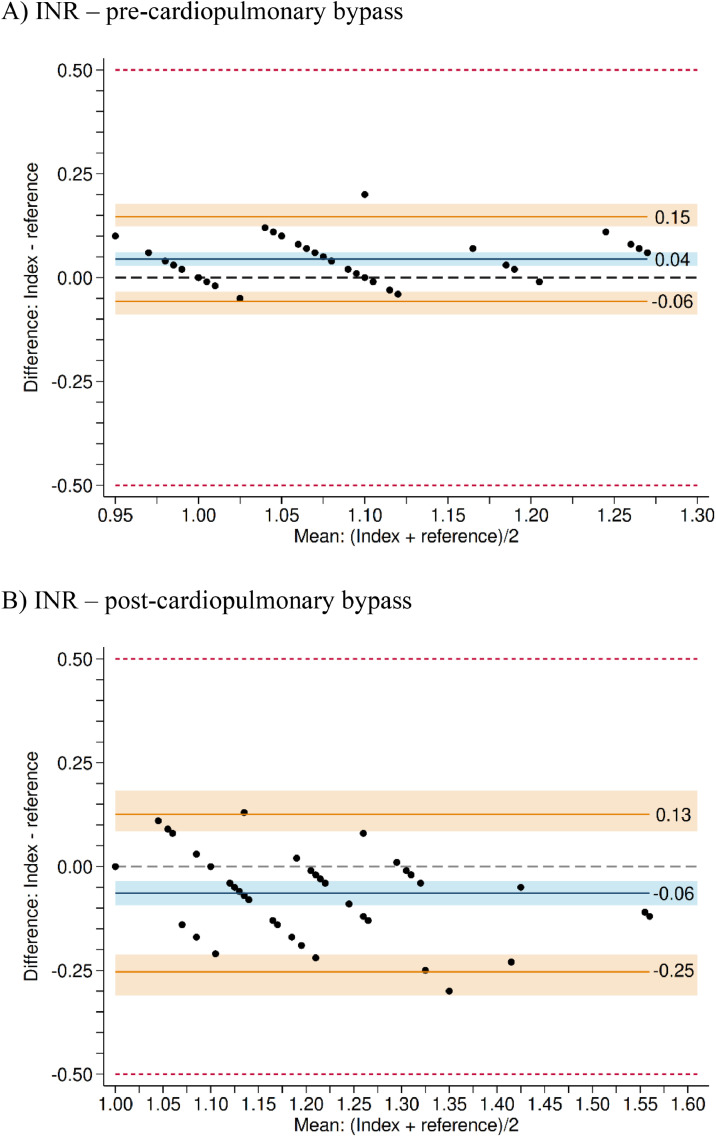
Agreement between the index and reference test for International Normalized Ratio (INR). Results are based on 50 participants. Points represent the measurements for each participant. The blue line represents the mean difference between the tests (in INR units). The orange lines represent the limits of agreement. It is expected that 95% of individuals will fall within these limits. The 95% Confidence Intervals are represented by the shaded areas (in blue and orange). The dashed line centered at zero (vertical axis) represents perfect agreement between the index and reference tests. When the blue region does not overlap with this line, we can conclude that the index test is associated with a statistically significant overestimation or underestimation relative to the reference test measurements. In panel A, a statistically significant overestimation is observed, while in panel B, a statistically significant underestimation is noted. The red dashed lines at -0.5 and 0.5 represent the pre-specified tolerance range. Differences within this tolerance range were not considered clinically relevant.

Figure 2 (panels A‒C) presents the Bland-Altman plots comparing the index and reference tests for INR, Prothrombin Time (PT), and Prothrombin Activity (PA) levels before cardiopulmonary bypass. Lin’s concordance correlation coefficients were 0.72 for INR (95% CI: 0.60 to 0.82), 0.66 for PT (95% CI: 0.51 to 0.78), and 0.69 for PA (95% CI: 0.57 to 0.80), indicating good concordance across all parameters. For INR, the index test showed a mean ± SD of 1.098 ± 0.089 and the reference test showed 1.053 ± 0.082, with a statistically significant difference in means (p < 0.001) but not in variability (p = 0.31). The mean difference was 0.045 units (95% CI: 0.030 to 0.059, p < 0.001), falling within the predefined tolerance range of ± 0.5 INR units. For PT, the index test showed a mean of 13.2 ± 1.10 seconds and the reference test 13 ± 1.53 seconds, with no significant difference in means (p = 0.21), although the variability differed statistically (p = 0.002). The mean difference was 0.2 seconds (95% CI: -0.11 to 0.51, p = 0.21), with limits of agreement ranging from -1.95 to 2.35 seconds. For PA, the index test showed a mean of 86% ± 11.4% and the reference test 92.7% ± 13%, with a statistically significant difference in means (p < 0.001), but not in variability (p = 0.12). Index test values were, on average, 6.65 percentage points lower than the reference (95% CI: -8.85 to -4.44, p < 0.001), falling within the Bland-Altman limits of agreement (-22.41 to 8.57 percentage points). For all three biomarkers, most participants had differences within the limits of agreement, with only a few measurements exceeding the boundaries: 2 participants (4%) for INR, 5 (10%) for PT, and 1 (2%) for PA.

### Agreement between the index and reference test – Post-cardiopulmonary bypass

**Figure 3:**
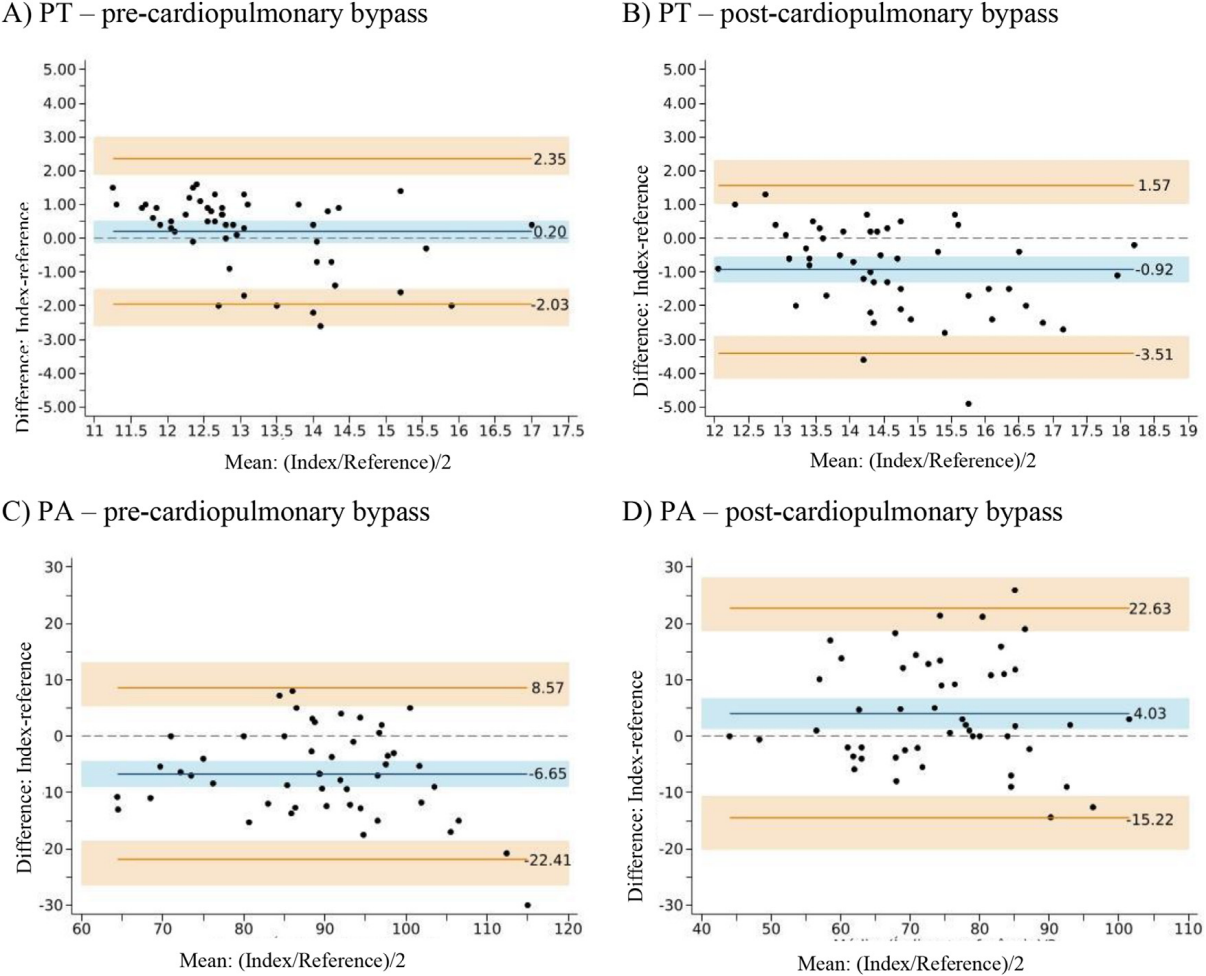
Secondary outcomes: Prothrombin Time (PT) and Prothrombin Activity (PA). All results are based on 50 participants. Comparison between the index test (CoaguChek) and the reference test (standard laboratory assay). Panels A and B show the results for PT (in minutes). Panels C and D show the results for PA (in percentage). Black dots represent the measurements for each of the 50 participants. The blue line represents the mean difference between the tests. The orange lines represent the limits of agreement. It is expected that 95% of patients will fall within these limits. The 95% Confidence Intervals are represented by the shaded areas (in blue and orange). The dashed line centered at zero (horizontal axis) represents perfect agreement between the index and reference tests. When the blue region does not overlap with this line, we can conclude that the index test shows a statistically significant overestimation or underestimation relative to the reference test measurements.

Figure 3 (panels A‒C) presents the Bland-Altman plots comparing the index and reference tests for INR, PT, and PA levels after cardiopulmonary bypass. Lin’s concordance correlation coefficients were 0.66 for INR (95% CI: 0.50 to 0.77), 0.55 for PT (95% CI: 0.37 to 0.69), and 0.70 for PA (95% CI: 0.54 to 0.82), indicating moderate to good concordance between the tests. For INR, the index test had a mean of 1.170 ± 0.116 and the reference test 1.234 ± 0.147, with statistically significant differences in both the mean (p < 0.001) and variability (p = 0.02). The average difference was -0.064 units (95% CI: -0.09 to -0.04, p < 0.001), falling within the tolerance range of ± 0.5 units. One participant (2%) exceeded the upper limit of agreement and 1 (2%) fell below the lower limit. For PT, the index test had a mean of 14.1±1.30 seconds and the reference test 15.1 ± 1.73 seconds. Both mean (p < 0.001) and variability (p = 0.008) differed statistically. The index test values were on average 0.92 seconds lower than those of the reference test (95% CI: -1.29 to -0.56, p < 0.001), with limits of agreement from -3.51 to 1.57 seconds. None of the participants exceeded the upper limit, while 2 (4%) fell below the lower limit. For PA, the index test had a mean of 76.4% ± 12.8% and the reference test 72.4% ± 13.3%; this difference was statistically significant (p = 0.004). There was no significant difference in variability (p = 0.73). On average, the index test measurements were 4.03 percentage points higher than those of the reference test (95% CI: 1.34 to 6.73, p < 0.001). Limits of agreement ranged from -15.22 to 22.63 percentage points. One participant (2%) exceeded the upper limit, and none fell below the lower limit.

## Discussion

### Main findings

In this prospectively planned, single-center, diagnostic accuracy study, we evaluated the accuracy of a point-of-care test for rapid assessment of the coagulation profile of patients undergoing cardiac surgery involving cardiopulmonary bypass. We found moderate to good agreement between the point-of-care and standard laboratory assays for the three coagulation markers examined (INR, PT, and PA). Although some statistically significant differences were observed between the index and reference test readings, they were not considered clinically significant and are unlikely to affect clinical decision-making.

### Comparison to previous studies

Previous studies have examined the agreement between point-of-care tests and conventional laboratory assays for rapid assessment of coagulation profiles in patients undergoing major surgery. These findings help contextualize our results.^15^^,^^16^

Our results are consistent with those reported by Urwyler et al. (2009), who assessed the accuracy of the CoaguChek in measuring prothrombin time compared with the standard laboratory assay in adult patients who underwent major surgeries. The device showed a sensitivity and specificity of 0.95, with an area under the ROC curve of 0.988, indicating equivalence between the point-of-care and laboratory tests. Overall, CoaguChek was considered a rapid and accurate method for intraoperative monitoring of prothrombin time in cases of suspected coagulopathy.^16^

Similarly, Meesters et al. (2016) assessed the agreement between the point-of-care CoaguChek and the standard laboratory assay in 50 adult patients undergoing cardiothoracic surgery with cardiopulmonary bypass. High agreement for INR values was observed before bypass, but discrepancies emerged at 3, 6, and 10 minutes post-protamine administration, with CoaguChek readings averaging 0.22 INR units lower than the standard laboratory assay. Nevertheless, the differences were not clinically significant for transfusion decisions, as INR discrepancies up to 0.5 are generally considered acceptable. These findings support the use of point-of-care testing in cardiac surgery and align closely with our results, reinforcing the value of rapid-result testing in this setting.^17^

In our study, although some comparisons reached statistical significance, the differences between CoaguChek and laboratory INR values remained within clinically acceptable limits and are unlikely to influence decision-making in practice. Given the sample size, statistical overpowering may have occurred, leading to the detection of statistical differences without clinical relevance. This highlights the importance of interpreting statistical findings within a real-world clinical context.

Okabayashi et al. (2018) also found that point-of-care devices such as the CoaguChek XS (Roche Diagnostics) provided clinically comparable results for PT and INR while enabling faster result acquisition in patients undergoing cardiac surgery and receiving warfarin therapy. Of note, the study indicated that heparin administration significantly prolonged PT/INR values in laboratory assays, with similar effects observed across different point-of-care devices, including the CoaguChek XS, Hemochron Jr., and DRIHEMATO PT (non-heparin neutralized). Both the CoaguChek XS and Hemochron Jr. use thromboplastin reagents with an ISI close to 1.0, resulting in a strong correlation with standard thromboplastin reagents used in clinical laboratories for plasma PT/INR. According to Okabayashi et al. (2018), PT/INR results obtained using the CoaguChek XS during cardiopulmonary bypass, in the range of 2.1 to 6.0, may overestimate the need for plasma transfusion. However, the number of participants with INR values above 2.0 was insufficient to draw definitive conclusions.^15^

From an economic point of view, CoaguChek appears to be cost-effective when compared with the standard laboratory assay. Hoel et al. evaluated the efficacy of the device in patients undergoing hemodialysis and found that the cost of the point-of-care test was lower than that of conventional laboratory tests.^18^ In addition, the time between test execution and result release is significantly shorter with the point-of-care device.

### Limitations

Our study has some limitations that should be acknowledged. First, although the sample size was determined based on a priori calculations to ensure sufficient statistical power for the primary outcome, the study was conducted in a single center with a specific patient profile, which may limit the external validity of the findings. Second, our statistical approach treated the pre- and post-cardiopulmonary bypass measurements as independent, rather than modeling them jointly. More sophisticated approaches, such as mixed-effects models, Deming regression, or Bayesian concordance models, could have been applied to assess agreement while accounting for repeated measures or measurement error in both tests. However, with only two observations per participant, these models would not provide additional value and could introduce instability or overfitting. Third, the time interval between the index (first) and reference tests was, on average, 30 minutes. Although this delay could have contributed to measurement differences, its impact was likely minimal due to the stability provided by the specialized collection tubes. Fourth, although viscoelastic tests are considered the gold standard for assessing coagulation in cardiac surgery,^19^ they were not included for comparison with CoaguChek in this study. The high cost and limited availability in many centers across the country were important limiting factors.

## Conclusion

CoaguChek XS demonstrated moderate to good agreement with standard laboratory assays for INR, PT and PA in adult patients undergoing elective cardiac surgery, supporting its clinical applicability for perioperative coagulation monitoring.

## Authors' contributions

Idealization: Sergio Honorato de Matos. Executing and writing: Larissa Goveia Moreira, Igor Louza Pereira, Lorenzo Leite Dino, Matheus Beserra Braga. Results: Fabrício Tavares Mendonça. Revision: Gustavo Henrique dos Santos Dias.

## Data availability statement

The datasets generated and/or analyzed during the current study are available from the corresponding author upon reasonable request.

## Conflicts of interest

The authors declare no conflicts of interest.

## Appendix Supplementary materials

Image, application 1

## Footnotes

Supplementary material associated with this article can be found in the online version at doi:10.1016/j.bjane.2025.844714.
