# Supplementary material for: Accuracy of a point-of-care CoaguChek test versus standard laboratory coagulation monitoring in cardiac surgery involving cardiopulmonary bypass: randomized clinical trial
Source: Braz J Anesthesiol. 2025 Nov 22;76(2):844714. doi: 10.1016/j.bjane.2025.844714 (PMC12828500; doi:10.1016/j.bjane.2025.844714)
Supplement: Supplementary file 1 [file mmc1.pdf]

Web-appendix 1

Anesthetic technique

Patients underwent anxiolysis with intravenous midazolam. Anesthesia induction was performed using fentanyl, lidocaine, etomidate, and rocuronium, while maintenance was achieved with inhaled sevoflurane. Mechanical ventilation was pressure-controlled with volume guarantee, with adjustments made to achieve an fraction of inspired oxygen of 0.4, inspiratory pressure sufficient to deliver a tidal volume of 6 mL/kg of ideal body weight, respiratory rate to maintain an end-tidal CO_2 (ETCO2) of 35-45 mmHg, and a positive end-expiratory pressure (PEEP) of 5 cmH_2O.

During cardiopulmonary bypass, the lungs were passively deflated. All patients underwent alpha-STAT cerebral protection, mild to moderate hypothermia (32–34°C), and mild hemodilution (hematocrit: 26–28%). Pump flow rates were set at 2.5 L/min/m² during the normothermic phase and 2.25–2.5 L/min/m² during the hypothermic phase, with a target mean arterial pressure of 70 mmHg. Intermittent antegrade blood cardioplegia was administered at an induction dose of 20 mL/kg, followed by maintenance doses of 10 mL/kg every 30 minutes.

At the end of the procedure, patients were transferred to the intensive care unit (ICU) under sedation with midazolam and controlled ventilation. The ICU team managed sedation weaning, mechanical ventilation, and inotropic support according to standard clinical protocols. We recorded the need for postoperative vasopressor and inotropic support, as well as the duration of mechanical ventilation for all patients.

Following anesthesia induction, a blood sample was collected to measure activated clotting time and blood gas parameters. From the same sample, international normalized ratio (INR), prothrombin time (PT) and and prothrombin activity were assessed using both standard laboratory methods and the CoaguChek device. These measurements were repeated 10 minutes after protamine administration, following the completion of cardiopulmonary bypass. All blood samples were drawn from venous access.

Web-appendix 2

A) INR - pre-Cardiopulmonary bypass

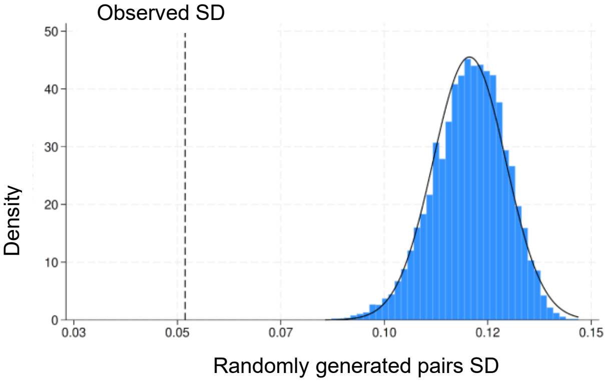

B) INR - post-Cardiopulmonary bypass

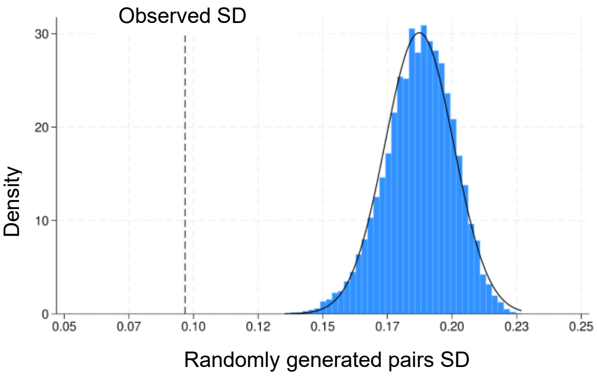

C) PT - pre-Cardiopulmonary bypass

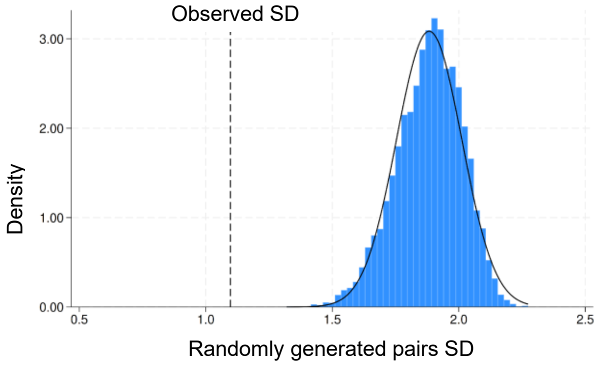

D) PT - post-Cardiopulmonary bypass

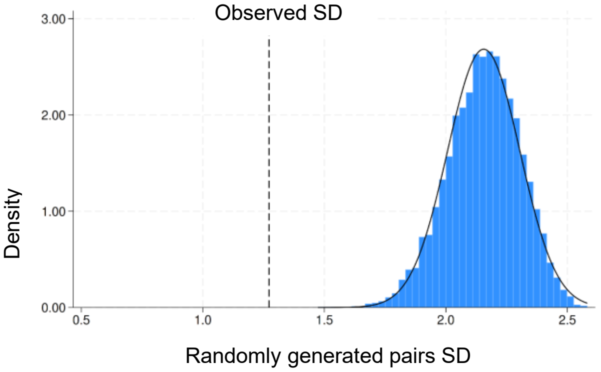

E) PA - pre-Cardiopulmonary bypass

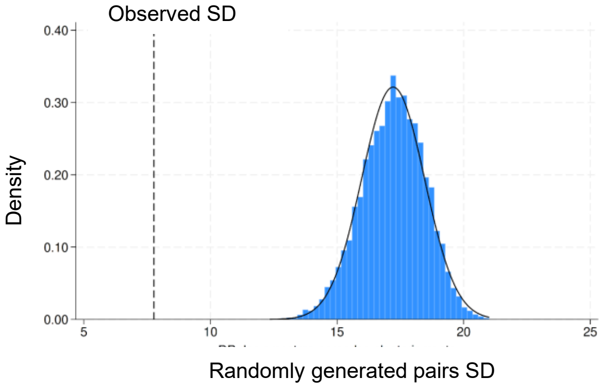

F) PA - post-Cardiopulmonary bypass

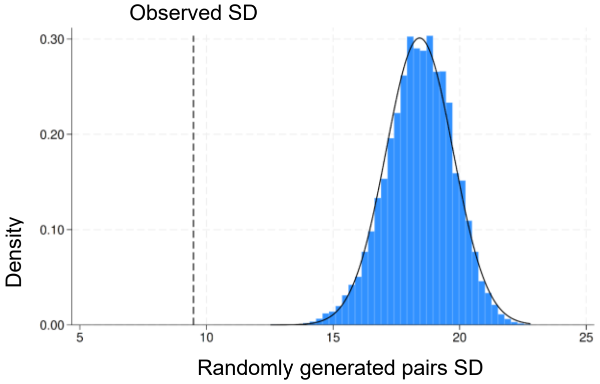

Web-figure 2. Distribution of standard deviations derived from 10,000 randomly generated pairs according to the Preiss-Fisher procedure (n = 50 participants). The observed standard deviation for each outcome is substantially lower than the minimum standard deviations.
